# RyR1 Is Involved in the Control of Myogenesis

**DOI:** 10.3390/cells14030158

**Published:** 2025-01-21

**Authors:** Amandine Tourel, Robin Reynaud-Dulaurier, Julie Brocard, Julien Fauré, Isabelle Marty, Anne Petiot

**Affiliations:** University Grenoble Alpes, Inserm, U1216, CHU Grenoble Alpes, Grenoble Institute Neurosciences, 38000 Grenoble, Francerobin.reynaud-dulaurier@univ-grenoble-alpes.fr (R.R.-D.); julie.brocard@univ-grenoble-alpes.fr (J.B.); julien.faure@univ-grenoble-alpes.fr (J.F.)

**Keywords:** calcium, myogenesis, RyR1

## Abstract

The RyR1 calcium release channel is a key player in skeletal muscle excitation–contraction coupling. Mutations in the *RYR1* gene are associated with congenital myopathies. Recently, a role of RyR1 in myotubes differentiation has been proposed and attributed to its calcium channel function, which nonetheless remains to be clearly demonstrated. In order to clarify RyR1 role in myogenesis, we have developed an in vitro model, the so-called RyR1-Rec myotubes, which are mouse primary myotubes with an inducible decrease in RyR1 protein amount and in RyR1-mediated calcium release. Using this model, we showed that the RyR1 protein decrease was responsible for an increase in both differentiation and fusion, from the RNA level to the morphological level, without affecting the myogenic factors MyoD and MyoG. Although an increase in mTOR pathway was observed in RyR1-Rec myotubes, it did not seem to be responsible for the role of RyR1 in myogenesis. Additionally, even if modulation of intracellular calcium level affected RyR1-Rec myotubes differentiation, we have shown that the role of RyR1 in myogenesis was independent of its calcium channel function. Therefore, our findings indicate that, besides its pivotal role as a calcium channel responsible for muscle contraction, RyR1 fulfills a calcium-independent inhibitor function of myogenesis.

## 1. Introduction

The type 1 ryanodine receptor (RyR1) is the main intracellular calcium channel in the skeletal muscle. RyR1 is localized in the sarcoplasmic reticulum (SR) membrane and constitutes a key component of excitation–contraction (EC) coupling machinery [1]. Upon depolarization of the transverse tubules, the activation of the RyR1 channel induces a huge calcium release from the SR into the cytosol, leading to muscle contraction [2]. Mutations in the *RYR1* gene can result in either a reduction in the protein expression or in functional alterations of the channel (gain-of-function or loss-of-function) [2,3] both of which disrupt cytosolic calcium homeostasis and are responsible for a spectrum of myopathies referred to as “RYR1-related myopathies”.

Myogenesis is a crucial biological process leading to muscle tissue formation during development and muscle regeneration following injury [4]. During the early phases of myogenesis, myogenic progenitor cells (myoblasts) can either proliferate or differentiate into myocytes, which subsequently fuse to form multinucleated muscle cells called primary myotubes. The growth of myotubes will then be ensured by fusion of primary myotubes with myoblasts [5]. The key players during the myogenesis process are a set of transcription factors, including Pax 3, Pax 7 and a group of muscle regulatory factors (MRF) composed of MyF5, MyoD, MyoG and MRF4 [6]. These four MRFs finely control the successive stages of differentiation, as Myf5 and MyoD are involved in myoblast determination and are followed by the activation of MyoG and MRF4, controlling myoblast differentiation. Myotubes differentiation is the complex result of intervention of those transcription factors as well as targeted proteins and signaling pathways, including among others, NOTCH, NFAT, Wnt/B catenin [7] and mTOR [8,9,10]. Moreover, myogenesis requires numerous proteins involved in fusion of muscle precursors, among which Myomaker (MymK) and Myomixer/Myomerger/Minion (MymX) are the main actors [11]. Additionally, calcium, essential for membrane fusion, plays an important role in myogenesis regulation [12].

The role of RyR1 in myogenesis has been recently studied using different tools, either pharmacological inhibitors or genetic tools. Indeed, inhibition of RyR1 calcium channel activity using its specific inhibitor dantrolene or reduction in RyR1 protein amount via siRNA or CRISPR/Cas-9 techniques led to alterations in myogenesis [13,14,15,16]. However, the precise role of RyR1 in myogenesis and the underlying mechanisms remain confused since both activator or inhibitor effects on myoblast differentiation were reported after modulation of the RyR1 protein or its calcium channel activity. In addition, most of these studies were carried out using the muscle cell line C2C12. To obtain results in a model closer to physiological development, we developed a primary myoblast model, the so-called RyR1-Rec myotubes, in which genetically induced reduction in RyR1 protein expression has been performed, followed by differentiation into myotubes. We first confirmed reduction in RyR1 protein amount and calcium release in RyR1-Rec myotubes. Using immunofluorescence and RT-qPCR, an increase in differentiation was observed in RyR1-Rec myotubes, suggesting an inhibitory function of RyR1 in myotubes differentiation. Moreover, our results suggest that this effect on muscle differentiation is independent of both the mTOR pathway and the RyR1 calcium channel activity. Our findings indicate that, in addition to its well-established role as a calcium channel responsible for muscle contraction, RyR1 fulfills a calcium-independent inhibitory function during myogenesis.

## 2. Materials and Methods

### 2.1. Antibodies and Reagents

The primary antibodies used in this study were polyclonal anti-RyR1 (described previously [17]), monoclonal anti-myosin heavy chain (MF20, DSHB Iowa city) and monoclonal anti-α1 subunit of DHPR (Abcam, Paris, France). Polyclonal anti-GAPDH, anti-mTOR, anti-Phospho mTOR, anti-S6 Ribosomal protein (5G10), anti-Phospho S6 Ribosomal protein (Ser240/244), anti-AMPK alpha, and anti-Phospho AMPK alpha (Thr 172) were from Ozyme (Ozyme, Saint Cyr l’Ecole, France). Secondary antibodies coupled to horseradish peroxidase (HRP) were from Jackson ImmunoResearch Laboratories (Jackson ImmunoResearch Laboratories, West Grove, PA, USA). Dantrolene, 4-Chloro-m-cresol (4-CmC), rapamycin and thapsigargin were purchased from Sigma–Aldrich (Sigma–Aldrich, St Louis, MO, USA).

### 2.2. Cell Culture

All procedures using animals were approved by the Institutional Ethics Committee and followed the guidelines of the National Research Council for the Care and Use of Laboratory Animals. Primary myoblasts from RyR1-Flox [18] newborn mice were prepared using a protocol adapted from Falcone et al. [19]. Briefly, after the isolation of hind limb muscles, the muscles were minced and digested for 15 min in IMDM, containing 1 mg/mL collagenase (Sigma-Aldrich, St Louis, MO, USA) and 7 mg/mL dispase (Life Technologies, Saint Aubin, France). After centrifugation, filtration and preplating to discard the contaminating fibroblasts, the myoblasts were amplified for 2–3 days in IMDM (Life Technologies) supplemented with 20% fetal bovine serum (FBS, Life Technologies), 1% penicillin/streptomycin (P/S, Life Technologies) and 1% chicken embryo extract (CEE, MP Biomedicals, Illkrich, France) before freezing. At the time of the experiment, the cells were seeded at 50,000 cells/cm^2^ on Matrigel (1/100, Corning Life Sciences, Corning, NY, USA) in a proliferation medium composed of HAM F-10 (Life technologies) supplemented with 20% FBS, 2% Ultroser (Sartorius, Göttingen, Germany) and 2% P/S. Twenty hours after seeding, differentiation was triggered by a switch to a differentiation medium composed of DMEM with glucose 1 g/L, 2% horse serum (Life Technologies, Saint Aubin, France) and 1% P/S.

### 2.3. Cell Treatment

Three types of cells have been used in this study: CTRL (non-transduced RyR1-Flox/Flox primary myoblasts), control DsRed cells (RyR1-Flox/Flox primary myoblasts transduced with AdV-DsRed, as a control of transduction effect) and RyR1-Rec cells (RyR1-Flox/Flox primary myoblasts transduced with AdV-Cre). For the production of control DsRed or RyR1-Rec myotubes, RyR1-Flox myoblasts were transduced with an adenovirus type 5 encoding the red fluorescent protein DsRed (AdV-DsRed kindly provided by Genethon, Evry, France) or the Cre recombinase (AdV-Cre, U.I. Viral Core, Iowa City, IA, USA) 4 h after cell seeding in proliferating medium at a multiplicity of infection (MOI) of 64. Twenty hours after seeding (considered as D0), differentiation was triggered by medium switch to differentiation medium. The differentiated myotubes were then used for analyses after 1 (D1), 2 (D2) or 3 days (D3) of differentiation. Rapamycin (final concentration 200 nM) was added at D1 or D2 until the collection of the myotubes at D3. Thapsigargin (final concentration 50 nM) and dantrolene (final concentration 20 µM) were added at D2 for 24 h.

### 2.4. Quantitative Real-Time PCR

Targeted gene expression was measured by quantitative real-time polymerase chain reaction (RT-qPCR). Total RNA was isolated from cell culture using SingleShot Cell Lysis Kit (Bio-Rad, Hercules, CA, USA) according to the manufacturer’s instructions. mRNA was reverse transcribed using iScript Reverse Transcription Supermix for RT-PCR (Bio-Rad, Hercules, CA, USA) according to the manufacturer’s instructions. RT-qPCR was performed using SsoAdvanced^TM^ Universal SYBR^®^ Green Supermix (Bio-Rad, Hercules, CA, USA). The primer sequences (forward “Fw” and reverse “Rv”) are listed in Table 1 and were designed using Primer designing tool from the NCBI. All reactions were run in triplicate on a CFX96 Touch^TM^ Real-Time PCR Detection System (Bio-Rad, Hercules, CA, USA) with the following amplification steps: 95 °C for 30 s; 39 cycles composed of 95 °C for 10 s, 60 °C for 20 s; 60 °C to 90 °C with increment of 0.5 °C every 5 s. The data were analyzed using CFX Maestro software v1.1 (Bio-Rad, Hercules, CA, USA). For each targeted transcript, expression was normalized to three housekeeping controls: *ACTB*, *HPRT* and *GAPDH* using the ΔΔC_t_ method.

### 2.5. Western Blot Analysis and Quantification

Myotubes lysates were collected in RIPA buffer (Tris-HCl 25 mM pH 7.6, NaCl 150 mM, NP-40 1%, sodium deoxycholate 1%, SDS 0.1%) containing a phosphatase inhibitors cocktail 2 (Sigma), PMSF (200 μM, Sigma) and DFP (1 mM, Sigma). The protein amount was quantified using Folin Lowry method, and 20 µg of protein was used for Western blot analysis performed as previously described [18]. Briefly, after electrophoretic separation on a 5–15% gradient acrylamide gel or a 10% acrylamide gel and electrotransfer to PVDF Immobilon P (Biorad, Hercules, CA, USA), the membrane was incubated with primary antibodies and then HRP-labeled secondary antibodies. Signal quantification was performed using a ChemiDoc Touch apparatus (Biorad, Hercules, CA, USA) and the Image Lab software v5.2.1 (Biorad, Hercules, CA, USA). The amount of the chosen protein in each sample was normalized to the amount of GAPDH or, for phosphorylated protein quantification, the total amount of the considered protein.

### 2.6. Calcium Imaging

Changes in intracellular calcium were measured on 3-day-old myotubes, as described previously [20], using the calcium-dependent fluorescent dye Fluo 4-Direct (Molecular Probes, Fisher Scientific, Illkirch, France) diluted in differentiation medium. Calcium imaging was performed in Krebs buffer (136 mM NaCl, 5 mM KCl, 2 mM CaCl_2_, 1 mM MgCl_2_, 10 mM HEPES, pH 7.4). KCl stimulation (140 mM final concentration) was performed by application of Krebs in which NaCl was replaced by KCl (140 mM KCl, 2 mM CaCl_2_, 1 mM MgCl_2_, 10 mM HEPES, pH 7.4). 4-CmC was applied at a 500 μM final concentration in Krebs and dantrolene at 20 µM. For some experiments, cultures were treated with dantrolene for 24 h before calcium imaging. Calcium fluorescence was recorded for 80 s, with an addition of drugs at 25 s. Thapsigargin was applied at 1 µM in calcium-free Krebs buffer (136 mM NaCl, 5 mM KCl, 3 mM MgCl_2_, 10 mM HEPES, pH 7.4, 1 mM EGTA, 0.1 mM LaCl_3_, 0.5 mM CdCl_2_), and calcium fluorescence was recorded for 300 s as described previously [20]. The curves represent the mean ± SEM of fluorescence variation after stimulation of the n myotubes in each condition, from at least three different experiments.

### 2.7. Immunofluorescence Staining

Cells cultures on FluoroDishes (World Precision Instruments, Friedberg, Germany) were fixed with 4% PFA in PBS for 20 min, treated for 10 min with NH_4_Cl before permeabilization in PBS supplemented with 1% Triton X-100 and saturated in PBS supplemented with 0.1% Triton X-100, 0.5% bovine serum albumin and 2% goat serum for 30 min. They were then incubated for 2 h at RT with primary antibodies in PBS supplemented with 0.1% Triton X-100, 0.5% bovine serum albumin and 2% goat serum. After 3 washes in PBS, fluorescent secondary antibodies were incubated for 1 h and the nuclei were labeled using Hoechst 33342 (Thermo scientific, Illkirch, France) before mounting with FluorSave™ reagent (Calbiochem, VWR, Rosny-sous-Bois, France). The images were captured on a Zeiss LSM710 confocal microscope. Imaging analysis was performed on 10 fields per dish (at least 3000 to 5000 cells) with 2 or more dishes/experiment and repeated at least on 4 independent cultures. The nuclei (total number and number in MF20^+^ cell) and MF20-labeled surface were automatically quantified using the ImageJ macro with a Stardist pluging. The number of myotubes was manually quantified. The area of each myotube (MF20^+^ surface divided by the number of myotubes), the number of nuclei per myotubes and the fusion index (the percentage of nuclei localized in MF20^+^ cell with at least 3 nuclei divided by the total number of nuclei) were subsequently calculated.

### 2.8. Statistics

The statistical analysis was performed using GraphPad Prism 6.0 software. The number of samples and the name of the parametric test applied are indicated in each figure legend. The results are considered significant when *p* < 0.05, the exact value for *p* being indicated in the figure legends, and significant results are labeled with a star (*) on the graphs regardless of the exact *p* value. All data are shown as mean ± SEM.

## 3. Results

### 3.1. Reduction in RyR1 Amount and Calcium Channel Activity in RyR1-Rec Primary Myotubes

**Figure 1:**
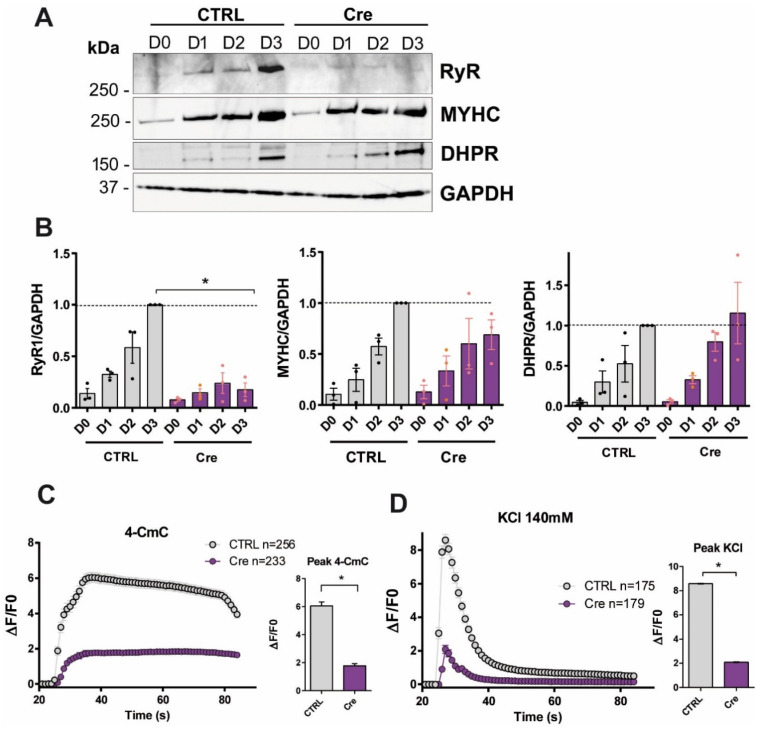
RyR1-Rec primary myotubes present a reduction in RyR1 amount and calcium release. (**A**) Representative Western blot of RyR1, MYHC and DHPR performed on non-treated (CTRL) and AdV-Cre treated (Cre) RyR1-Flox primary cell culture collected at different days of differentiation (D0 to D3). GAPDH is used as a loading control. (**B**) Quantification of the relative amount of each protein compared to GAPDH (CTRL, gray bars and RyR1-Rec, purple bars). All the data are presented as mean ± SEM of three different cultures. The mean value at D3 in the CTRL group was set to 1 as normalization for each protein. Statistical analysis: one sample *t*-test D3-Cre vs. D3. *p* = 0.0065 for RyR1. *, *p* < 0.05. (**C**,**D**) Fluo-4 calcium imaging performed on D3 myotubes produced from CTRL and RyR1-Rec culture (Cre). The curves represent the fluorescence variation in CTRL myotubes (gray curve) and RyR1-Rec myotubes (purple curve). All values are presented as mean ± standard error of mean (SEM) of n myotubes. In each condition, *n* = 175 to 256 myotubes have been analyzed, from at least three different experiments (exact number indicated for each curve). *, *p* < 0.05 (**C**) Kinetics of calcium release upon stimulation by 4-CmC at 500 µM. The stimulation is performed at 25 s and the fluorescence variation (ΔF/F0) recorded for 1 min. The peak amplitude of each curve is presented on the bar graph on the right. Statistical analysis: unpaired *t*-test, *p* < 0.0001. (**D**) Kinetics of calcium release upon stimulation by KCl at 140 mM. The peak amplitude of each curve is presented on the bar graph on the right. Statistical analysis: unpaired *t*-test, *p* < 0.0001.

To better understand the potential role of RyR1 in muscle cells differentiation, we developed an in vitro model in order to decrease RyR1 protein levels in primary mouse myotubes. Satellite cells were isolated from new-born RyR1-flox mice [18], and the alteration of *RYR1* gene expression in those cells was induced by transduction with an adenovirus encoding the Cre recombinase (AdV-Cre) that allowed recombination between RyR1 loxP sites and the ablation of *RYR1* expression. Control cells (CTRL) and AdV-Cre-transduced cells (so called RyR1-Rec or Cre) were induced in differentiation for 3 days and further used for different analysis. Whereas in control cells, RyR1, myosin and DHPR progressively increased from the first to the the third day of differentiation (Figure 1A,B) as expected, RyR1-Rec cells presented a decrease in RyR1 protein amount, compared to CTRL since the first day of differentiation (Figure 1A,B). After 3 days of differentiation, an 80% decrease in RyR1 protein amont was observed in RyR1-Rec myotubes compared to CTRL myotubes. This decrease in RyR1 amount in RyR1-Rec myotubes was not associated with modification in myosin or DHPR amounts. We then checked the effect of the decrease in RyR1 protein amount on intracellular calcium release. Calcium imaging experiments were realized and, as expected, decrease in RyR1 amount in primary myotubes cultures (RyR1-Rec) was associated with a decrease in calcium release induced by the RyR1 agonist 4-CmC (Figure 1C) as well as by KCl-induced membrane depolarization (Figure 1D). Taken together, these results show that RyR1-Rec primary myotubes presented a decrease in RyR1 protein associated with a decrease in RyR1-related calcium release. Thus, these myotubes constitute a useful tool to better understand the role of a decrease in RyR1 protein expression in muscle cell differentiation.

### 3.2. Reduction in RyR1 Amount in Primary Myotubes Is Associated with an Increase in Myotubes Differentiation Without Affecting Myogenic Factors

**Figure 2:**
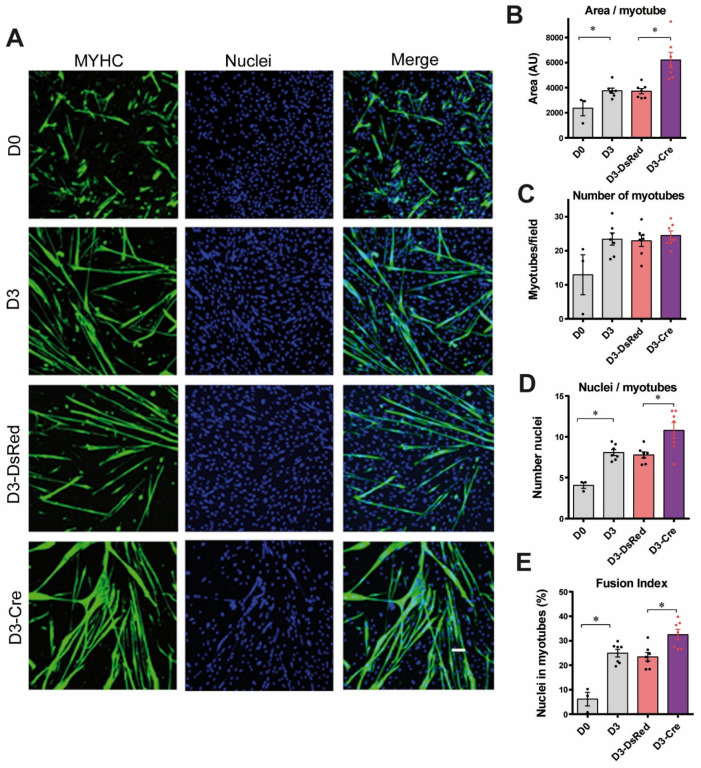
The reduction in RyR1 protein amount in primary myotubes is associated with an increase in myotube differentiation. (**A**) Representative immunofluorescence images of cultures in proliferation (D0) and after 3 days of differentiation, non-transduced (D3) or transducted with AdV-DsRed (D3-DsRed) or AdV-Cre (D3-Cre). The myotubes were stained with myosin heavy chain (MYHC, green) and the nuclei with Hoechst (blue). Scale bar: 100 um. (**B**) Quantification of the area of myotube, defined as the total surface occupied by myotubes divided by the number of myotubes per field, in at least 20 fields for each condition. Statistical analysis: Mann–Whitney *t*-test. D3 vs. D0 *p* = 0.0167; D3-Cre vs. D3-DsRed *p* = 0.0006. *, *p* < 0.05. (**C**) Quantification of the number of myotubes per field. Statistical analysis: Mann–Whitney *t*-test, non-significant. (**D**) Quantification of the number of nuclei per myotube, from at least five different cultures. Statistical analysis: Mann–Whitney *t*-test. D3 vs. D0 *p* = 0.0167, D3-Cre vs. D3-DsRed *p* = 0.0379. *, *p* < 0.05. (**E**) Quantification of fusion index, representing the percentage of nuclei inside MYHC-positive cells with three or more nuclei/total nuclei. Statistical analysis: Mann–Whitney *t*-test. D3 vs. D0 *p* = 0.0167, D3-Cre vs. D3-DsRed *p* = 0.0041. *, *p* < 0.05.

Surprisingly, even though we did not observe obvious modifications in the amount of myosin and DHPR, two markers of myotube differentiation, in RyR1-Rec myotubes (Figure 1A,B), immunofluorescence analysis clearly showed alteration in the morphology of these RyR1-Rec myotubes (Figure 2A). In order to further dissect the role of RyR1 in myotubes differentiation, an in-depth characterization of 3 day-differentiated RyR1-Rec myotubes was performed (Figure 2B–E).

**Figure 3:**
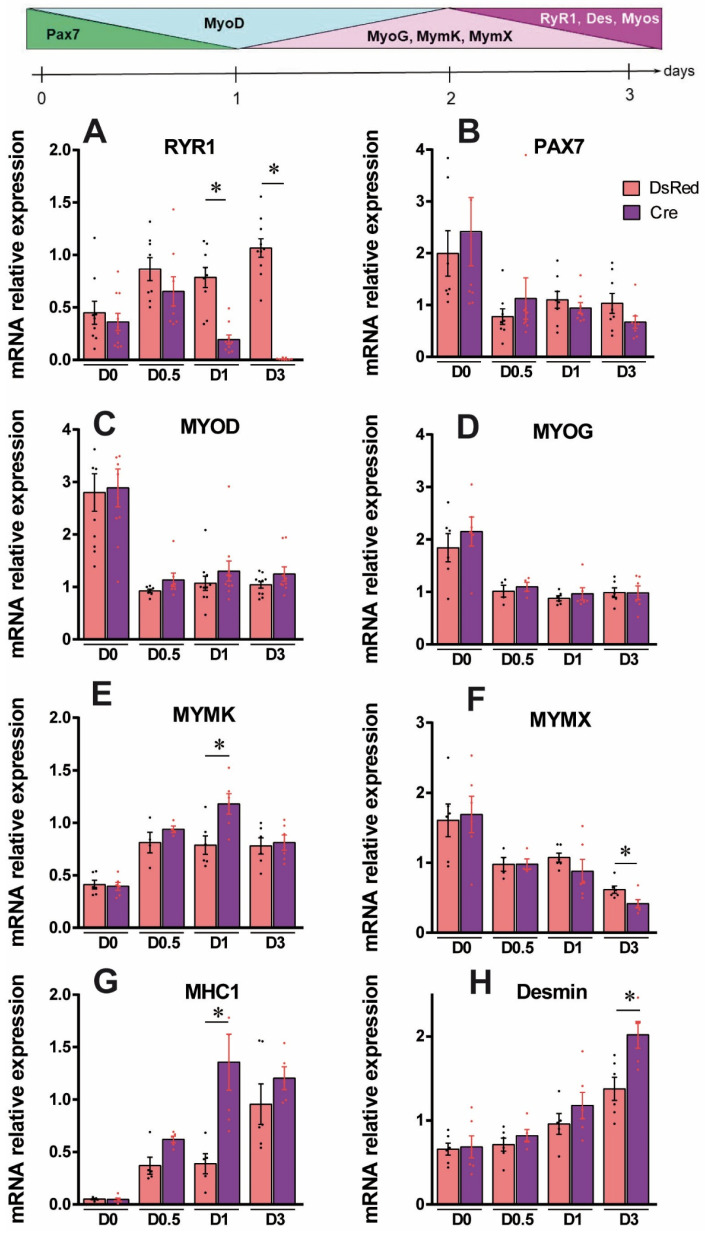
Reduction in *RYR1* mRNA in primary myotubes is associated with an increase in late marker of myotube differentiation. The mRNA levels of myoblast proliferation/differentiation markers were quantified in control (DsRed, red bars) and RyR1-Rec (Cre, purple bars) cultures at different time points after the induction of differentiation (D0) up to 3 days (D3) by quantitative RT-qPCR. (**A**) Relative amount of *RYR1* mRNA. Statistical analysis: Mann–Whitney comparison of RyR1-Rec vs. DsRed at each time point. On D1, *p* = 0.0002; on D3, *p* < 0.0001. *, *p* < 0.05. (**B**) Relative amount of *PAX7* mRNA. Statistical analysis: Mann–Whitney comparison of RyR1-Rec vs. DsRed at each time point. Non-significant difference at each time. (**C**) Relative amount of *MYOD* mRNA. Statistical analysis: Mann–Whitney comparison of RyR1-Rec vs. DsRed at each time point. Non-significant difference at each time point. (**D**) Relative amount of *MYOG* mRNA. Statistical analysis: Mann–Whitney comparison of RyR1-Rec vs. DsRed at each time point. Non-significant difference at each time point. (**E**) Relative amount of *MYMK* mRNA. Statistical analysis: Mann–Whitney comparison of RyR1-Rec vs. DsRed at each time point. On D1, *p* = 0.0152. *, *p* < 0.05. (**F**) Relative amount of *MYMX* mRNA. Statistical analysis: Mann–Whitney comparison of RyR1-Rec vs. DsRed at each time point. At D3 *p* = 0.0411. *, *p* < 0.05. (**G**) Relative amount of myosin heavy chain *MHC1* mRNA. Statistical analysis: Mann–Whitney comparison of RyR1-Rec vs. DsRed at each time point. On D1, *p* = 0.0043. *, *p* < 0.05. (**H**) Relative amount of Desmin. Statistical analysis: Mann–Whitney comparison of RyR1-Rec vs. DsRed at each time point. On D3 *p* = 0.0303. *, *p* < 0.05.

Control (CTRL i.e., RyR1-flox) myoblasts in proliferating medium (D0) or after 3 days of differentiation (D3) were compared to RyR1-flox transduced with a non-relevant adenovirus Adv-DsRed, followed by 3 days of differentiation (D3-DsRed), or RyR1-flox transduced with Adv-Cre, followed by 3 days of differentiation (D3-Cre). Compared to the D0 condition, the myotube area, number of nuclei per myotubes (Figure 2B–D), and the fusion index (Figure 2E) were increased in D3, D3-DsRed as well as D3-Cre myotubes, thus validating the induction of myoblasts differentiation. No modification in those different parameters was observed between the D3 and D3-DsRed myotubes, demonstrating that the transduction of the primary cultures with a non-relevant adenovirus did not modify the differentiation of the cultures. In contrast, after 3 days of differentiation, the cultures treated with Adv-Cre (i.e., RyR1-Rec) were characterized by an increase in myotube area compared to control cultures (D3-Cre versus D3 and D3-DsRed). Although the total number of myotubes was not modified between D3-Cre and D3-DsRed cultures (Figure 2C), the number of nuclei per myotube as well as the fusion index (Figure 2D,E) were significantly increased in D3-Cre, showing that the decrease in RyR1 protein level was associated with an increase in myotubes differentiation. In order to confirm this result, RT-qPCR was used to follow the expression of different markers of myoblast differentiation during myogenesis. Pax 7 expression was used as a marker of satellite cells. It reflects the proliferative status of myoblasts. The two myogenic factors, MyoD (a marker of the early phase of differentiation) and MyoG (a marker of the middle phase of differentiation), were used in order to evaluate MRFs involvement, whereas MHC1 (encoding myosin-heavy chain) and desmin were used as markers of the late phase of differentiation. Moreover, myomaker (MymK) and myomixer (MymX) were used to characterize the fusion stage during muscle cell differentiation. Quantification at each time point, presented in Figure 3, shows that in control primary myotubes (DsRed), *RYR1*-mRNA amount increased continuously as soon as 12 h after differentiation induction (Figure 3A). In contrast, in RyR1-Rec myotubes, a decrease in *RYR1*-mRNA was observed since the first day of differentiation and no *RYR1* mRNA was present after 3 days of differentiation, in line with the reduced amount of RyR1 protein observed in RyR1-Rec myotubes, as seen in Figure 1. *PAX7*-mRNA, *MYOD*-mRNA and *MYOG*-mRNA were at maximal level on D0 and decreased rapidly to reach a stable level over 3 days (Figure 3B–D), both in control and RyR1-Rec myotubes. No difference in *PAX7*-mRNA, *MYOD*-mRNA and *MYOG*-mRNA levels was observed at any time between control cultures and RyR1-Rec cultures, suggesting that the reduction in *RYR1*-mRNA was not associated with a modification of cell proliferation or the expression of MRFs (Figure 3B–D). *MYMK*-mRNA increased 12 h after the induction of differentiation and remained stable until D3 in control cells (Figure 3E). However, it presented a peak on D1 in RyR-Rec compared to control before returning to similar level on D3. *MYMX*-mRNA had a maximum level on D0 and slowly decreased until D3 (Figure 3F), both in control and in RyR-Rec, with a more pronounced decrease on D3 in RyR1-Rec. *MHC1*-mRNA increased progressively from the beginning of differentiation in control as well as RyR1-Rec myotubes, but more rapidly in RyR1-Rec, with a large difference on D1 (Figure 3G). Desmin-mRNA increased progressively after 24 h (D1) in differentiation medium, in control as well as RyR1-Rec myotubes, but faster in RyR1-Rec, thus leading to a large difference on D3 (Figure 3H). Taken together, these results clearly demonstrate that the decrease in RyR1 was associated with an increase in the myotubes differentiation at 3 days. Moreover, the role of RyR1 in muscle differentiation was associated with an increase in markers of the late phase of differentiation and/or fusion, without impact on the MRFs.

### 3.3. RyR1 Quantity and Calcium Channel Activity Modulate the mTOR Pathway

**Figure 4:**
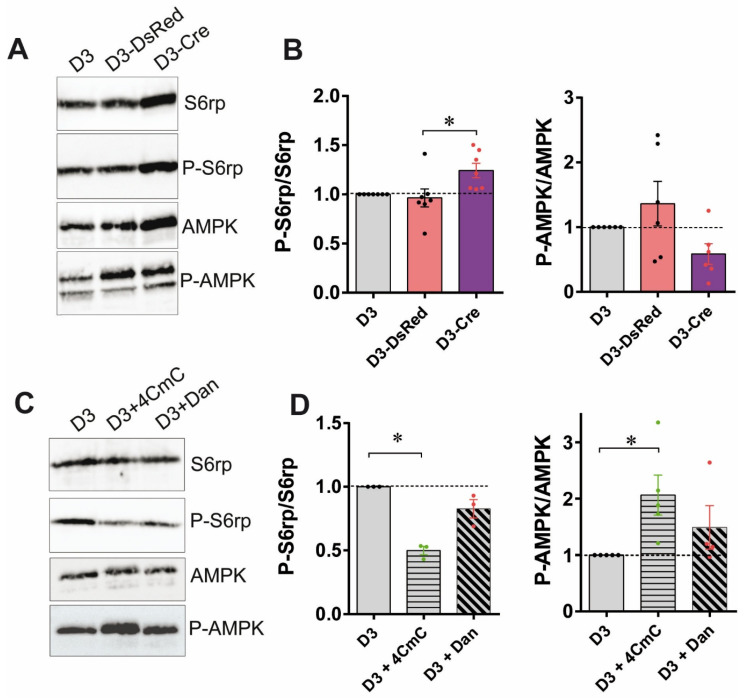
Both RyR1 quantity and calcium channel activity modulate the AMPK/mTOR pathway. (**A**) Representative Western blot of total and phosphorylated forms of S6rp and AMPK proteins on control (D3), AdV-DsRed transduced (D3-DsRed) and AdV-Cre transduced (D3-Cre, i.e., RyR1-Rec) RyR-Flox primary cell cultures at 3 days of differentiation. (**B**) Quantification of the relative amount of phosphorylated S6rp compared to the total S6rp amount performed in five to six independent cultures (left graph), and the relative amount of phosphorylated AMPK compared to the total AMPK amount performed in five to six independent cultures (non-transduced CTRL D3, gray bars; D3-DsRed, red bars; D3-Cre, purple bars). The amount in non-transduced cells is set to 1 for normalization. Statistical analysis: Mann–Whitney. D3-Cre vs. D3-DsRed *p* = 0.0111 for P-S6rp/S6rp. *, *p* < 0.05. (**C**) Representative Western blot of total and phosphorylated forms of S6rp and AMPK proteins on differentiated myotubes either non-treated (D3), treated for 3 h at 3 days of differentiation with 4-CmC (D3 + 4CmC), or treated for 3 h with Dantrolene (D3 + Dan). (**D**) Quantification of the relative amount of phosphorylated S6rp compared to the total S6rp amount performed in four independent cultures (left graph), and the relative amount of phosphorylated AMPK compared to the total AMPK amount performed in independent cultures (non-treated D3 myotubes, gray bars; myotubes treated with 4CmC horizontal stripes, myotubes treated with Dantrolene inclined stripes). The amount in non-treated cells is set to 1 for normalization. Statistical analysis: Mann–Whitney. D3 + 4CmC vs. D3 *p* = 0.0043 for P-S6rp/S6rp; D3 + 4CmC vs. D3 *p* = 0.0407 for P-AMPK/AMPK. *, *p* < 0.05.

The Mammalian Target of Rapamycin (mTOR) signaling network has been described as a modulator of skeletal myogenesis [8], and we previously reported an increase in the mTOR pathway in RyR1-Rec mice [18]; therefore, we investigated the status of the mTOR pathway in our primary mouse cultures. The phosphorylation state of the S6 ribosomal protein (S6rp), a downstream effector of mTOR whose phosphorylation reflects its activity, was analyzed in control myotubes (D3 and D3-DsRed) and RyR1-Rec myotubes (D3-Cre). As observed on the representative Western blot (Figure 4A), mock-transduction of the primary cultures with Adv-DsRed (D3-DsRed) did not modify the total S6rp and the phosphorylated S6rp protein (P-S6rp), and, overall, the ratio P-S6rp/S6rp compared to the non-transduced cultures (D3) was not modified (Figure 4B), suggesting no direct effect of the viral transduction on mTOR activity. But the reduction in RyR1 protein (D3-Cre) was associated with an upregulation of both total S6rp and phosphorylated S6rp (Figure 4A) in RyR1-Rec myotubes compared to control myotubes (D3 and D3-DsRed). It also resulted in an increase in the ratio of P-S6rp/S6rp (Figure 4B) in RyR1-Rec myotubes, which suggested a hyperactivation of the mTOR pathway in myotubes with reduced RyR1. As the mTOR pathway could be inhibited by AMP kinase (AMPK) [21], the activity of AMPK was analyzed in RyR1-Rec myotubes. A decrease in the phosphorylation state of AMPK (P-AMPK) was associated with an increase in total AMPK protein (AMPK) (Figure 4A), leading to an overall decrease in P-AMPK/AMPK being observed, pointing to a decrease in AMPK activity (Figure 4B). Although this effect did not reach significance (*p* = 0.0931), as AMPK is an inhibitor of mTOR activity, this result correlates with the increase in mTOR activity observed in RyR1-Rec myotubes. These data suggest that the decrease in RyR1 protein was associated with an activation of the mTOR pathway, probably mediated by inhibition of the AMPK. To further determine if the RyR1-mediated Ca^2+^ release can affect the mTOR pathway, control myotubes were treated with the RyR1 agonist 4-CmC (3 h at 500 µM) to induce an increase in cytosolic calcium. This treatment was clearly associated with a decrease in mTOR activity, reflected by a decrease in P-S6rp/S6rp ratio and an increase in P-AMPK/AMPK ratio (Figure 4C,D). The application of the RyR1-inhibitor dantrolene, (3 h at 20 µM), which was not associated with any observable release of calcium into the cytoplasm (Figure S1A), had no significant effect on P-S6rp/S6rp or P-AMPK/AMPK ratios (Figure 4C,D). Taken together, these results showed that the decrease in the RyR1 protein amount was associated with an activation of the mTOR pathway. Conversely, activation of RyR1-mediated calcium release was associated with a decrease in the mTOR pathway via the control of the AMPK pathway, suggesting a role of RyR1 calcium release in the AMPK/mTOR pathway.

### 3.4. The Increased Myotube Differentiation in RyR1-Rec Myotubes Is Independent of the mTOR Pathway

**Figure 5:**
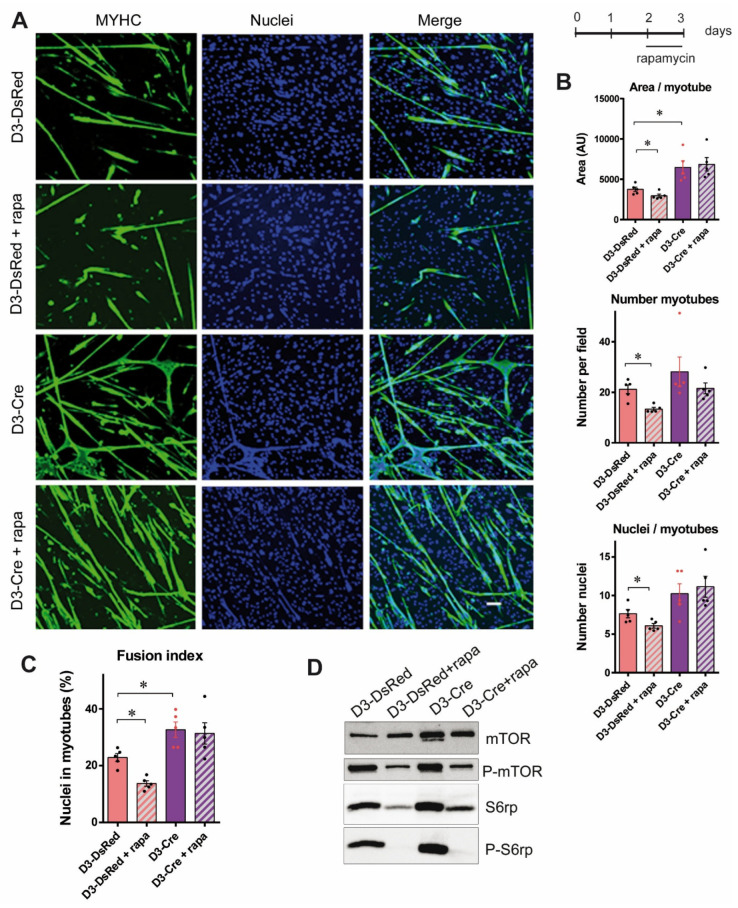
The increased myotube differentiation in RyR1-Rec myotubes is independent of the mTOR pathway. (**A**) Representative immunofluorescence images of myotubes at 3 days of differentiation from control (D3-DsRed) or RyR1-Rec (D3-Cre) cultures, treated or untreated with rapamycin (200 nM) for the last 24 h of differentiation. The myotubes were visualized with myosin heavy chain staining (MYHC, green) and the nuclei with Hoechst (blue). Scale bar: 100 μm. (**B**) The mean area of myotube, number of myotubes per field, and number of nuclei by myotubes in control myotubes (D3-DsRed) or RyR1-Rec myotubes (D3-Cre), treated or untreated with rapamycin (24 h, 200 nM), from five independent cultures. Statistical analysis: Mann–Whitney *t*-test. Myotubes area D3-DsRed + rapa vs. D3-DsRed *p* = 0.0317; D3-Cre vs. D3-DsRed *p* = 0.0079. Number of myotubes D3-DsRed + rapa vs. D3-DsRed *p* = 0.0159; nuclei/myotubes D3-DsRed + rapa vs. D3-DsRed *p* = 0.0317. *, *p* < 0.05. (**C**) Quantification of fusion index, representing the percentage of nuclei inside MYHC-positive cells with three or more nuclei/total nuclei on five independent cultures. Statistical analysis: Mann–Whitney *t*-test. D3-DsRed + rapa vs. D3-DsRed *p* = 0.0079; D3-Cre vs. D3-DsRed *p* = 0.0079. *, *p* < 0.05. (**D**) Representative Western blot showing total and phosphorylated forms of mTOR and S6rp, in control (D3-DsRed) and RyR1-Rec (D3-Cre) 3-day-myotubes with or without rapamycin treatment (24 h, 200 nM).

Since we observed an activation of the mTOR pathway induced by the reduction in RyR1 protein amount, and as reduction in RyR1 expression was associated with an increase in myotube differentiation, we assessed if the effect of RyR1 reduction on myotube differentiation could be mediated by activation of the mTOR pathway. We, therefore, treated cultures on D2 with the mTOR inhibitor rapamycin for 24 h and evaluated the different parameters related to myotube differentiation. For the control cells, the treatment of DsRed myotubes with rapamycin induced a decrease in the myotube area, number of myotubes, number of nuclei per myotube (Figure 5A,B) as well as reduction in fusion index (Figure 5C), compared to non-treated DsRed myotubes, (D3-DsRed + rapa compared to D3-DsRed), thus confirming that the inhibition of mTOR pathway was associated with a decrease in fusion index in control cells. The inhibitory effect of rapamycin on mTOR and S6rp phosphorylation in different cultures was confirmed with Western blot (Figure 5D). But in RyR1-Rec myotubes, neither the area of the myotube, the total number of myotubes, the number of nuclei per myotube (Figure 5A,B), nor the fusion index (Figure 5C) were modified by rapamycin treatment compared to non-treated RyR1-Rec cultures (D3-Cre+ rapa compared to D3-Cre). These data suggest that the myotube differentiation increase in RyR1-Rec was not dependent of mTOR activity.

The mTOR activity has been described in C2C12 to have an oscillating behavior, with a decrease at the induction of differentiation followed by a progressive increase during the differentiation of myotubes [22]. Therefore, to ascertain that the mTOR inhibition has been performed at the optimal timing of differentiation in our model, an earlier rapamycin treatment was tested. The myotubes were treated on D1 of differentiation for 48 h before analysis (Figure S2). The fusion index was not modified by the earlier treatment (Figure S2B), neither in control (D3-DsRed-Rapa versus D3-DsRed) nor in RyR1-Rec myotubes (D3-Cre + rapa versus D3-Cre), although the rapamycin treatment effectively decreased the mTOR and S6rp phosphorylation statuses in control and in RyR1-Rec cultures (Figure S2C).

Taken together, our results show that the increase in myotube differentiation observed in RyR1-Rec myotudes was independent of the mTOR pathway.

### 3.5. RyR1 Function on Myotube Differentiation Is Independent of RyR1-Mediated Calcium Release

**Figure 6:**
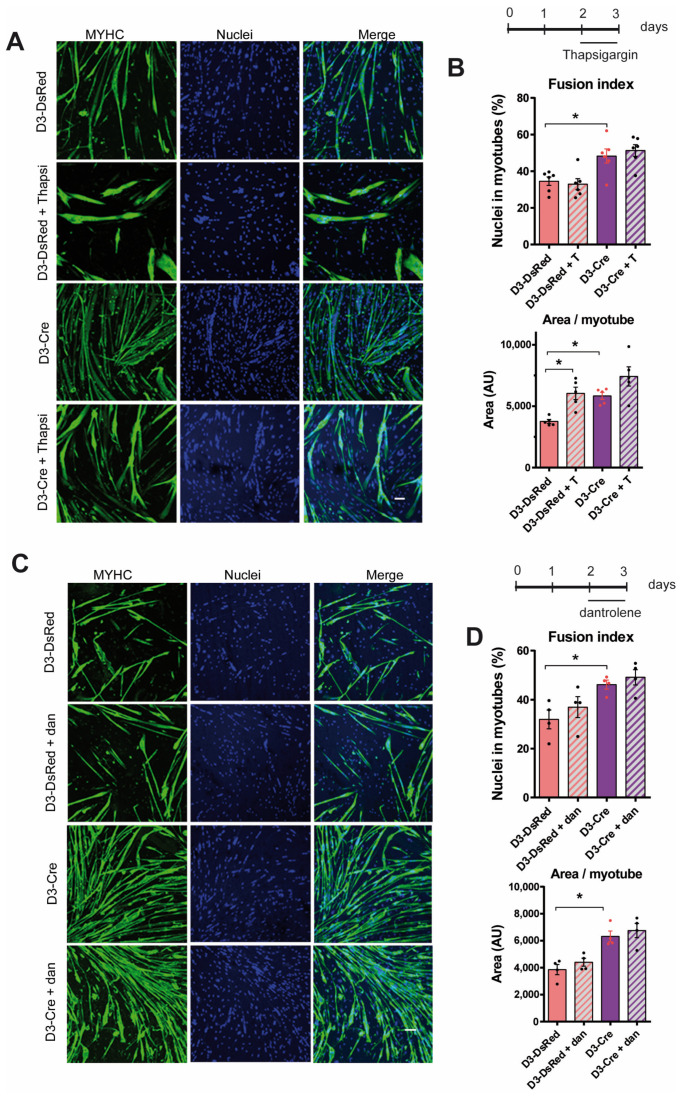
RyR1 effect on myotube differentiation is independent of RyR1-mediated calcium release. (**A**) Representative immunofluorescence images of primary myotube culture at 3 days of differentiation from control (D3-DsRed) or RyR1-Rec (D3-Cre) cultures and treated or untreated with thapsigargin (Thapsi or T, 50 nM) for the last 24 h of differentiation. The myotubes were visualized with myosin heavy chain staining (MYHC, green) and the nuclei with Hoechst (blue). Scale bar: 100 μm. (**B**) The fusion index and area of myotubes in control myotubes (D3-DsRed) or RyR1-Rec myotubes (D3-Cre), treated or untreated with thapsigargin (24 h, 50 nM), from five to six independent cultures. Statistical analysis: Mann–Whitney *t*-test. Fusion index: D3-Cre vs. D3-DsRed *p* = 0.0260. Area of myotubes: D3-DsRed + Thapsi vs. D3-DsRed *p* = 0.0079; D3-Cre vs. D3-DsRed *p* = 0.0079. *, *p* < 0.05. (**C**) Representative immunofluorescence images of primary myotubes culture at 3 days of differentiation from control (D3-DsRed) or RyR1-Rec (D3-Cre) cultures, treated or untreated with dantrolene (20 µM) for the last 24 h of differentiation. The myotubes were visualized with myosin heavy chain staining (MYHC, green) and the nuclei with Hoechst (blue). Scale bar: 100 μm. (**D**) The fusion index and area of myotubes in control myotubes (D3-DsRed) or RyR1-Rec myotubes (D3-Cre), treated or untreated with dantrolene (48 h, 20 µM), from four independent cultures. Statistical analysis: Mann–Whitney *t*-test. Fusion index: D3-Cre vs. D3-DsRed *p* = 0.0286. Area of myotubes: D3-Cre vs. D3-DsRed *p* = 0.0286. *, *p* < 0.05.

Since it has been proposed that increase in intracellular calcium was required for myotubes differentiation and/or fusion [14,16] due to a ryanodine receptor-induced Ca^2+^ release, we tested if an alteration in Ca^2+^ release could be responsible for the alteration in myotubes differentiation observed in RyR1-Rec myotubes. The SERCA pump was blocked using thapsigargin, subsequently inducing an increase in the cytosolic calcium due to the uncompensated calcium leak from the SR. We first confirmed that the acute application of thapsigargin resulted in a cytosolic calcium increase in the same amplitude in control and RyR1-Rec myotubes (Figure S1B), as calcium leak from the SR following inhibition of re-uptake was proposed to be, at least in part, due to RyR1 [23] and, therefore, could be modified by RyR1-deletion. Surprisingly, chronic 24 h thapsigargin (50 nM) treatment did not induce a modification of the fusion index, either in control myotubes (D3-DsRed and D3-DsRed + T; Figure 6A,B) or in RyR1-Rec myotubes (D3-Cre and D3-Cre + T; Figure 6A,B). However, as can be seen in Figure 6A, treatment of myotubes with thapsigargin clearly induced an alteration of the overall myotubes morphology, which resulted in an increase in myotube area in control myotubes (D3-DsRed + T vs. D3-DsRed) but not in RyR1-Rec myotubes (D3-Cre + T vs. D3-Cre). These data confirm the implication of Ca^2+^ in myotube organizations and suggest that intracellular calcium increase was not responsible for the increase in myotube differentiation observed in RyR1-depleted myotubes. Finally, in order to assess specifically the influence of RyR1 Ca^2+^ release on differentiation, we treated the different cultures with dantrolene, a specific RyR1 inhibitor. Dantrolene treatment (24 h at 20 µM) decreased RyR1 calcium release in control myotubes (DsRed) upon stimulation (Figure S1C) without modification in the fusion index or the area of the myotubes in control. In RyR1-Rec myotubes, dantrolene treatment had no effect on calcium release, as expected, due to the absence of RyR1 (Figure S1C), and thus did not induce modification in the fusion index or the area of the myotubes (Figure 6C,D). Overall, our data show that calcium channel activity of RyR1 was not involved in myotube differentiation.

## 4. Discussion

The involvement of ryanodine receptors in myogenesis has long been studied, and the ryanodine receptor isoform RyR3 [24] has been proposed to play a major function during muscle differentiation. But more recently, besides its role in muscle contraction, RyR1 has been clearly involved in muscle differentiation, both in the C2C12 cell model [14,15] and in vivo [25]. However, the precise role of RyR1 in myogenesis remains unclear, as contrasting results have been reported depending on the tools and culture conditions used [14,15]. To further investigate RyR1’s function in myogenesis, we developed the first in vitro model of primary mouse myoblasts with an inducible RyR1 depletion, the RyR1-Rec myoblasts. Using these myoblasts, we demonstrated that RyR1 acts as a negative regulator of myogenesis, as its inhibition significantly increases differentiation. These findings are in line with a previous study showing that treatment of C2C12 myoblasts with siRNA, targeting RyR1-induced myoblast hyperfusion [15]. In contrast, our findings challenge a previous report suggesting that RyR1 inhibition or reduced calcium release activity was associated with decreased myotube differentiation [14]. Both the amount and timing of RyR1 expression during myogenesis may be key to RyR1 regulation of differentiation, which could explain the apparent discrepancies between the different studies. Therefore, we propose that there could be a threshold of RyR1 amount, below which activation of differentiation is observed, and above which inhibition is induced. In addition, the precise timing of RyR1 inhibition, compared to myotube formation/muscle differentiation, could also be of major importance on the final effect on differentiation, as the precise timing of myotube differentiation is different between the C2C12 cell line, primary cultures or in vivo in mice. This question would require additional studies.

In the present study, we did not observe any effect of RyR1 reduction in the MRFs MyoD and MyoG, which could be related to the strong commitment of our cell model in differentiation. Most probably, the effect of RyR1 on these factors could take place even before D0 of our study, the primary cultures being already activated cells compared to the C2C12 cell line, as observed by the expression of RyR1 and other myotube-specific proteins on D0.

Besides the effect of RyR1 protein amount on myogenesis modulation, the direct involvement of calcium release in myogenesis has been studied by pharmacological modulation. Pharmacological regulation could lead to complex results, as different intracellular calcium channels are expressed in skeletal muscle cells. The ryanodine receptors (RyR1 and RyR3) and IP3 receptors (IP3R) work in complex interplay to control intracellular calcium concentration spatially and temporarily [26,27]. As Ca^2+^-signaling plays a central role in myogenesis [12] and, more specifically, during cell fusion [28], it has been proposed that RyR1’s involvement in myogenesis could be related to its calcium channel activity. But contrasting results have been obtained. Some studies have reported that inhibiting RyR1’s calcium release (e.g., with dantrolene treatment) reduced cytosolic calcium and was associated with decreased myotube differentiation [15,16]. But other studies have suggested a decreased myotube differentiation and fusion associated with an increased intracellular calcium release through RyR1 due to the loss of caveolin-3 [29], and low concentration of intracellular calcium was required for myo-lineage commitment in pig primary muscle cells [30]. In our experiments, although increasing intracellular calcium through thapsigargin treatment did not alter the fusion index, it clearly affected myotube morphology. Moreover, as the RyR1 inhibitor dantrolene did not alter the differentiation in control or RyR1-Rec myotubes, it demonstrated that RyR1-mediated calcium release was not required during myogenesis in primary culture cells. Even if the intracellular calcium is important for myotubes formation and does not require RyR1-calcium channel function, it may be due to other calcium channels, such as RyR3 or IP3R. However, using RT-qPCR, we observed that RyR1 deletion was not associated with an up-regulation of *RYR3* in 3- day RyR1-Rec myotubes (Figure S3), excluding a RyR3 compensation to explain the increased differentiation in RyR1-Rec myotubes. The involvement of other calcium channels would require additional studies.

Besides an effect on intracellular calcium, RyR1 function in myogenesis could be related to its effect on endoplasmic reticulum (ER) stress. Indeed, ER stress has been observed in association with RyR1 reduction [14,15], and ER stress has been proposed to influence myoblast differentiation and fusion [31,32,33]. ER stress can be induced by multiple factors in addition to Ca^2+^ depletion, such as misfolded proteins accumulation, oxidative stress, or glucose deprivation. It also results in altered gene expression and differentiation. The activation of the unfolded protein response (UPR) pathway, a cellular stress response related to ER stress, is essential for myogenesis [34,35]. The Inositol-requiring transmembrane kinase endoribonuclease 1 alpha (IRE1α)/X-Box Binding Protein 1 (XBP1) (IRE1α/XBP1) pathway, one of the three arms involved in UPR, has been shown to be required for myotube differentiation [36,37] and myoblast fusion [38]. Along this line, reduction in IRE1α was associated with reduction in myotube differentiation [36,37], and it has been shown recently that deficiency of IRE1α or XBP1 in satellite cells specifically inhibits myoblast fusion without having any effect on the myogenic differentiation program. Moreover, IRE1α/XBP1 was proposed to specifically regulate myomaker gene expression [38]. Interestingly, RyR1 depletion in C2C12 myoblasts led to hyperfused myotubes and ER stress without significant changes in ER Ca^2+^ content [15]. So, even if the IRE1α/XBP1 has not yet been explored in RyR1-Rec, an interesting hypothesis would be an RyR1/IRE1α/XBP1/MymK link, which could explain the role of RyR1 in myogenesis observed in RyR1-Rec myotubes. Such an RyR1/IRE1α link and UPR activation have been observed in the RyR1-I4895T mouse model, with a mutant RyR1 leading to misfolded SR proteins [39]. Therefore, in our model, the absence of RyR1 could result to the unfolding of some RyR1 partners in the macromolecular calcium release complex (such as DHPR, triadin, calsequestrin [2]), resulting in UPR activation, an increase in IRE1α-linked MymK activation, and enhanced differentiation.

In addition to its well-established function in the regulation of cell and muscle growth, an important role of mTOR signaling has been identified in myogenesis [8]. It has been proposed that the initiation of differentiation and myotube formation are controlled by mTOR independently of its kinase activity, whereas myotube growth and maturation require mTOR kinase activity [40]. We previously reported an increase in the mTOR pathway in the muscles of RyR1-Rec mice [18], and confirmed here an increase in mTOR activity in myotubes depleted of RyR1 protein. But no correlation could be established between the mTOR modulation and myogenesis modification, since the mTOR inhibitor rapamycin did not affect differentiation in RyR1-Rec myoyubes. AMPK, an upstream mTOR inhibitor that plays a crucial role in maintaining metabolic homeostasis [41,42], is involved in many cellular functions and processes, including energy metabolism, protein synthesis, proliferation, differentiation, autophagy and apoptosis [43]. It has been shown that AMPK activation blocks muscle proliferation and differentiation [44,45,46,47,48]. Moreover, AMPKα2 has been recently reported as an intrinsic regulator of myonuclear accretion, a key step in myotube formation, in skeletal muscle stem cell [49]. Along this line, the potential modification of the AMPK pathway that we observed in RyR1-Rec myotubes differentiation would require further investigations.

## 5. Conclusions 

Overall, this study describes the first in vitro primary mouse model with a decreased level of RyR1. Our results pointed to an inhibitory role for RyR1 in muscle cells differentiation and fusion, independent of both the mTOR pathway and the RyR1 calcium channel activity. Our findings indicate that, in addition to its well-established role as a calcium channel responsible for muscle contraction and strength, RyR1 fulfills a calcium-independent inhibitory function in myogenesis.

## Figures and Tables

**Table 1:** Primer for RT-q-PCR.

Primers	Sequence 5′-3′
*RYR1*_Fw	TGTTTGACCATCTCCCCTTCTG
*RYR1*_Rv	AAGGAAGTAGCCTTGGTGTGG
*PAX7*_Fw	AGGATGATGAGACCCGGCCC
*PAX7*_Rv	GGTCGACCGTTGATGAAGACCC
*MYOD*_Fw	ACTCTCACGGCTTGGGTTGAGG
*MYOD*_Rv	TCGGGGCCTGTCAAGTCTATGTC
*MYOG*_Fw	TTGCTCAGCTCCCTCAACCAGG
*MYOG*_Rv	GAGGCGCTGTGGGAGTTGCA
*MHC1*_Fw	CTGCACCAGCTGAGGTGTAA
*MHC1*_Rv	TCTAGGAGCCCCAGAAGACC
*Desmin*_Fw	AGGCTCAAGGCCAAACTACAGGAG
*Desmin*_Rv	ATCCACATCCGCTCGGAAGG
*MYMK*_Fw	CGTGACATTCTGGAGTACTTCAGCATC
*MYMK*_Rv	GAAGGTCGATCTCTGGGGTTCATC
*MYMX*_Fw	GTTAGAACTGGTGAGCAGGAG
*MYMX*_Rv	CCATCGGGAGCAATGGAA
*ACTB*_Fw	CTAAGGCCAACCGTGAAAAG
*ACTB*_Rv	ACCAGAGGCATACAGGGACA
*GAPDH*_Fw	CGTGCCGCCTGGAGAAAC
*GAPDH*_Rv	TGGGAGTTGCTGTTGAAGTCG
*HPRT*_Fw	CCTAATCATTATGCCGAGGATTTGG
*HPRT*_Rv	CCCATCTCCTTCATGACATCTCGAG
*RYR3*_Fw	CCACTGAGCTGGTCCACTTT
*RYR3*_Rv	GGGTTCGTCCTTGCCGATAA

## Data Availability

The data presented in this study are contained within the article or Supplementary Material.

## Supplementary Materials

The following supporting information can be downloaded at: https://www.mdpi.com/article/10.3390/cells14030158/s1, Figure S1: Effect of acute or chronical treatment of the myotubes on calcium release; Figure S2: 48h rapamycin treatment does not affect myotube differentiation in primary myotubes cultures; Figure S3: Evolution of RyR3 mRNA in primary myotubes.
