# Supplementary material for: RyR1 Is Involved in the Control of Myogenesis
Source: Cells. 2025 Jan 21;14(3):158. doi: 10.3390/cells14030158 (PMC11817019; doi:10.3390/cells14030158)

Supplementary data

Figure S1: Effect of acute or chronical treatment of the myotubes on calcium release.
Changes in intracellular calcium were measured on 3 day-control primary myotubes using the calcium-dependent fluorescent dye Fluo 4-AM. Data are given as mean ± S.E.M of n myotubes. **A)** At 25s (arrow), calcium release has been induced by application of 4-CmC (500µM, red curve) or dantrolene (20µM, yellow curve). The acute application of the RyR1 inhibitor dantrolene didn't induce calcium release. Application of the RyR1 agonist 4-CmC induced a huge calcium release. **B)** At 25s (arrow), calcium release has been induced by application of thapsigargin (1µM) in control (DsRed, red curve) or RyR1-Rec myotubes (Cre, purple curve), and fluorescence variation recorded for 5 min. Calcium release of the same amplitude was observed in the two cultures. **C)** Chronical treatment of the myotubes with dantrolene altered 4-CmC induced-calcium release in control myotubes but not in RyR1-Rec myotubes. The control (DsRed) or RyR1-Rec (Cre) myotubes were treated for 24h with dantrolene (dan, 20µM) before calcium imaging. At 5s (arrow), the myotubes were stimulated by 4-CmC (500µM) and fluorescence variation recorded for 60 s.The dantrolene treatment resulted in reduction of calcium release upon stimulation in control (DsRed) myotubes, but has no effect in RyR1-Rec myotubes (Cre), confirming a dantrolene action only in the presence of RyR1.

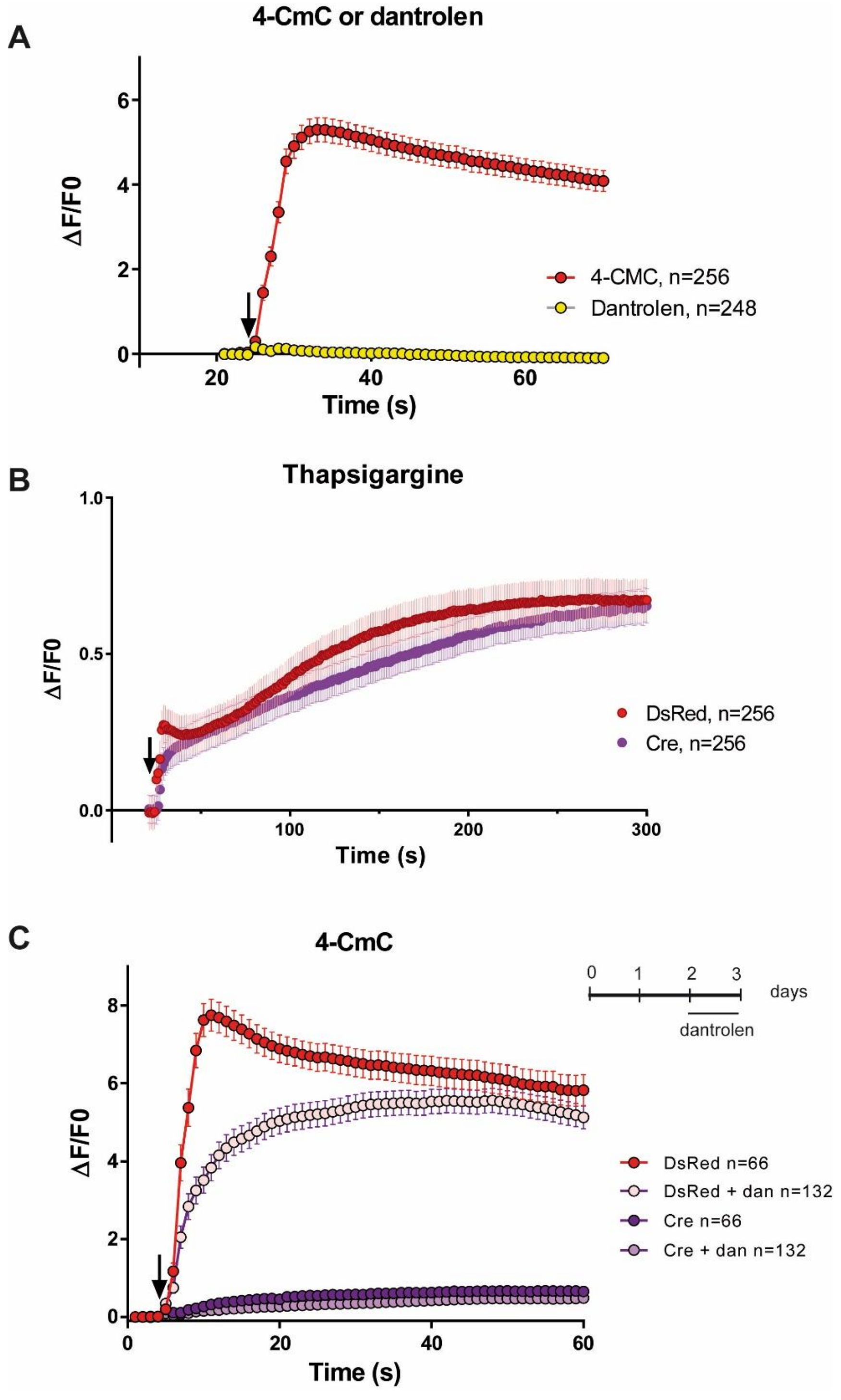
A
4-CmC or dantrolen
ΔF/F0
Time (s)
4-CMC, n=256
Dantrolen, n=248
B
Thapsigargine
ΔF/F0
Time (s)
DsRed, n=256
Cre, n=256
C
4-CmC
ΔF/F0
Time (s)
0 1 2 3
days
dantrolen
DsRed n=66
DsRed + dan n=132
Cre n=66
Cre + dan n=132

Figure S2: 48h rapamycin treatment does not affect myotube differentiation in primary myotubes cultures: A) Representative immunofluorescence images of 3 day-control myotubes (D3-DsRed) and RyR1-Rec myotubes (D3-Cre) treated or not with rapamycin (200 nM) for the 48 last hours of differentiation. Cells were stained with myosin heavy chain antibody (MYHC, green) and the nuclei with Hoechst (blue). Scale bar: 100µm. **B)** Fusion index in control myotubes (D3-DsRed) or RyR1-Rec myotubes (D3-Cre), treated or not with rapamycin (rapa, 48h, 200 nM), from 4 independent cultures. Statistical analysis: Mann Whitney t-test. D3-Cre *vs* D3-DsRed p=0.0286. **C)** Representative western blot showing total and phosphorylated forms of mTOR and S6rp, on DsRed and Cre (RyR1-Rec) 3 days myotubes with or without rapamycin treatment (rapa 48h, 200 nM).

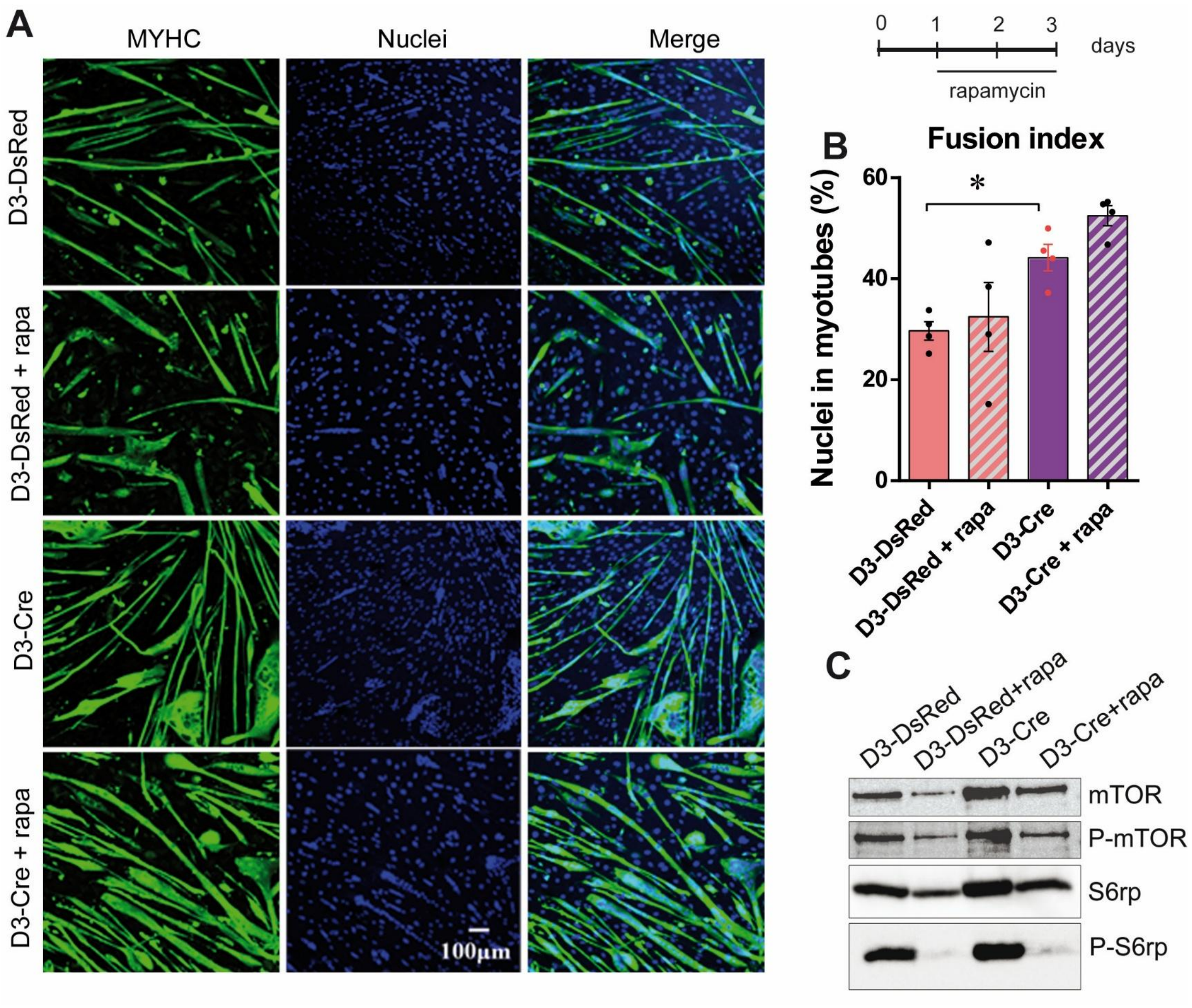

Figure S3: Evolution of RyR3 mRNA in primary myotubes. mRNA level of RYR3 was quantified in control (DsRed, red bars) and in RyR1-Rec (Cre, purple bars) cultures at different time points after induction of differentiation (D0) up to 3 days (D3), by quantitative RT-qPCR. Statistical analysis: Mann Whitney t-test at each time of DsRed *vs* Cre; at D1 p=0.0411.

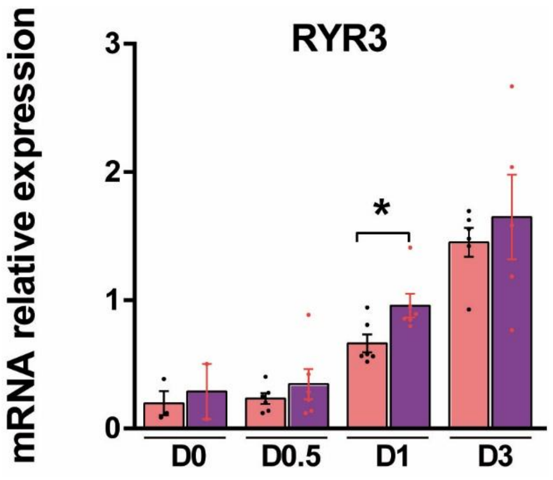

Supplement: Supplementary file 1 [file cells-14-00158-s001.zip › cells-3399976-supplementary.pdf]
